# Effectiveness of School‐Based Nutrition Interventions in Italy: A Scoping Review

**DOI:** 10.1002/fsn3.70266

**Published:** 2025-07-15

**Authors:** Stephen Gambescia, Basil H. Aboul‐Enein, Teresa Keller, Fatmah Almoayad, Nada Benajiba

**Affiliations:** ^1^ College of Nursing and Health Professions Drexel University Philadelphia Pennsylvania USA; ^2^ College of Arts and Sciences, Health & Society Program University of Massachusetts Dartmouth North Dartmouth Massachusetts USA; ^3^ Faculty of Public Health and Policy London School of Hygiene and Tropical Medicine London UK; ^4^ School of Nursing New Mexico State University Las Cruces New Mexico USA; ^5^ College of Health and Rehabilitation Sciences, Department of Health Sciences Princess Nourah Bint Abdulrahman University Riyadh Saudi Arabia; ^6^ Joint Research Unit in Nutrition and Food Ibn Tofail University—CNESTEN Kenitra Rabat Morocco

**Keywords:** children, Italy, nutrition, school

## Abstract

School‐based nutrition interventions play a crucial role in promoting healthy eating habits among children and adolescents, which are fundamental for preventing chronic diseases and fostering physical and mental development. This scoping review aims to examine the literature on the nature and extent of research conducted in school‐based nutrition intervention programs in Italy and their effectiveness. Established and emerging programs and practices are reviewed to identify successful strategies in Italy. A scoping review of published studies using the PRISMA‐ScR guidelines across 14 databases for school‐based nutrition interventions implemented in Italy was conducted, and the results communicate literature published from 1990 to December 2024. The review included 42 studies, highlighting positive changes in dietary knowledge and behavior, with 33 studies reporting significant improvements. Interventions employing a sequence of education (knowledge), demonstration by teachers, and practice of good nutrition at home and/or school demonstrated sustained behavioral changes. Each study was conducted by a high number of authors, showing healthy collaboration. Studies were mainly applied using a Health Promotion Model, without reference to theories. However, gaps remain in addressing adolescent populations, employing social marketing techniques, and integrating environmental changes. While Italian schools have implemented diverse and culturally tailored interventions, challenges persist, including variability in program implementation, limited follow‐up, and underutilized theoretical frameworks. Efforts to enhance collaboration among schools, families, and policymakers are essential for sustainable success. School‐based nutrition interventions in Italy have demonstrated substantial potential in improving dietary behaviors among children and adolescents. Addressing identified gaps and promoting comprehensive, multicomponent approaches can further enhance the long‐term success of these programs.

## Graphical abstract

This scoping review aims to examine the literature on the nature and extent of research conducted in school‐based nutrition intervention programs in Italy and their effectiveness. Established and emerging programs and practices are reviewed to identify successful strategies in Italy. While Italian schools have implemented diverse and culturally tailored interventions, challenges persist, including variability in program implementation, limited follow‐up, and underutilized theoretical frameworks. Regional distribution of school‐based nutrition intervention studies in Italy (North, Central, and South).

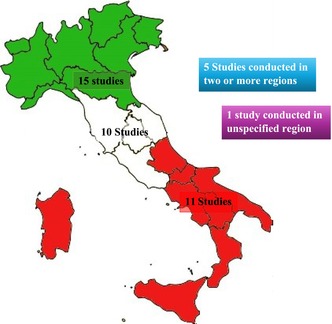

## 1 Introduction

Healthy eating habits among school‐age children are the basis for strong mental and physical development as adults. Healthy eating habits promote growth and reduce many risks associated with chronic diseases such as obesity, diabetes, and cardiovascular disease. A robust body of research literature conducted globally supports the importance of nutrition education offered through schools. However, while the importance of nutrition education has been established, a comprehensive understanding of the best school nutrition interventions and programs is still not fully understood (CDC 2024). School nutrition and effective interventions that encourage healthy eating habits continue to be a significant focus for researchers in school health (Al‐Jawaldeh et al. 2023; WHO 2021).

In Italy, with the growing burden of the childhood obesity crisis (Binkin et al. 2010; Palermi et al. 2022; Silano et al. 2019; Spinelli et al. 2023), school nutrition interventions have been used to promote healthier eating habits among children, leveraging the country's rich culinary traditions. These interventions are often based on the Mediterranean diet (MD), renowned for its health benefits and cultural significance (Aureli and Rossi 2022; Buja et al. 2020). The MD is characterized by high consumption of fruits, vegetables, whole grains, legumes, nuts, olive oil, fish, and poultry (Deleu et al. 2024). This diet has been associated with numerous health benefits, including reduced risks of cardiovascular diseases, diabetes, and obesity. This dietary pattern fits well with school nutrition programs' goals to foster long‐term healthy eating habits in children (Martíncrespo‐Blanco et al. 2022).

Sociocultural factors profoundly influence school nutrition programs, especially the influence of family and cultural eating habits on young children. Encouraging children to adopt healthier eating habits can be difficult if these habits are not reinforced at home (Buja et al. 2020; Sanmarchi et al. 2022; Sanmarchi et al. 2023). Meals are seen as important social events, and family preferences heavily influence children's eating habits (Costanzo 2011). Although the traditional Italian diet is generally considered to be healthy, Italian cuisine includes regional variations and traditional foods that may not always align with modern nutritional guidelines.

Sociocultural impacts on food choices vary significantly between Northern and Southern Italy. The North is more industrialized and offers a higher standard of living. The surrounding countries of Switzerland, Austria, and France have a cultural impact on lifestyle and cuisine in Northern Italy. Southern Italy is more rural, with an economy dependent on agriculture. The climate and proximity to two oceans provide a diet rich in vegetables, olive oil, and fish and are influenced by other countries that border the Mediterranean Sea (Grosso et al. 2013). These differences mean that national school nutrition programs must consider these differences when crafting regionally sourced programs acceptable to local schoolchildren.

Other challenges to school nutrition programs include external forces that affect the sustainability of a school nutrition program. The disruption experienced during the COVID‐19 pandemic was created when schools were closed and the routine acquisition and delivery of food resources was interrupted (Ferrero et al. 2023; Vitali et al. 2023). School nutrition programs are affected by state and national regulatory oversight of public school feeding programs (Loperfido et al. 2024; Marcotrigiano et al. 2021). Addressing the external forces that negatively impact school nutrition programs requires coordinated efforts at national, regional, and local levels to ensure that all children have access to nutritious meals and effective nutrition education (McIsaac et al. 2018). Addressing these sociocultural influences requires a holistic approach that involves not only schools but also families, communities, health communication outlets, popular culture, and policymakers. By understanding and addressing the broader social and cultural context, school nutrition programs can be more effective in promoting healthy eating habits among children. This scoping review examines the literature on the nature and extent of school‐based nutrition interventions in Italy and their effectiveness. Established and emerging programs and practices are reviewed to identify successful strategies in Italy. By synthesizing existing research, this review seeks to provide insights into the impact of these interventions on children's dietary behaviors and health outcomes in Italy and areas for further study on types of students, schools they attend, and novel areas to explore.

## 2 Methods

### 2.1 Selection Criteria

The population, intervention, comparison, outcomes, and study (PICOS) design guidelines (Higgins et al. 2019) were incorporated to develop the research question: “Do school‐age students in Italy (P) that are offered school‐based nutrition interventions (I) have improved health and wellness parameters (O) compared with those that do not participate in school‐based nutrition interventions(C)?” and subsequent inclusion and exclusion criteria (see Table 1). Peer‐reviewed articles published in English were included. Interventions reported outside traditional peer‐reviewed articles were excluded in this review. The search was conducted in the winter of 2024 and the results communicate literature published from 1990 to December 2024.

**TABLE 1:** PICOS criteria for inclusion and exclusion of studies.

Parameter	Inclusion criteria	Exclusion criteria
Population	School‐aged students (i.e., around 6 years old and above) who were examined in Italy	Students who are not of school age. Students who are not studying in Italy. Students undergoing medical nutrition therapy‐based diets.
Intervention type	Any kind of school‐based intervention that addresses nutrition‐related aspects, including: Educational interventions Environmental interventions Multicomponential interventions	Interventions that are not based in any part of school facilities Interventions that do not address nutrition‐related outcomes
Comparators	Preintervention, baseline nutrition‐related variables (i.e., anthropometric measures, biochemical parameters, nutrition‐related knowledge, dietary habits, perceived hunger) of student groups who were as follows: Control: received no intervention. Received partial intervention, e.g., educational intervention only vs. multicomponential intervention.	N/A
Outcomes of interest	Changes in anthropometric outcomes, e.g., BMI for age, height for age Changes in biochemical outcomes Changes in nutrition‐related knowledge Changes in meeting the dietary macronutrient and/or micronutrient recommendations Changes in adherence to healthy dietary habits and avoidance of unhealthy ones Changes in dietary habits concomitant with physical activity Decrease in risks of nutrition‐related diseases, e.g., obesity or iron‐deficiency anemia Decrease in short‐term hunger	Non‐nutrition‐related outcomes
Study type	Intervention studies with measurable outcomes	Non‐numeric/categorical assessments or qualitative studies
Language	English or Italian	All other languages
Study type	Peer‐reviewed original research intervention articles Original research conference intervention publications	Non–peer‐reviewed articles Commentaires Narratives Communications Nonintervention‐based studies White papers Similar article types Gray literature

Abbreviations: BMI: body mass index; N/A: not applicable.

### 2.2 Search Procedures

A scoping review of the literature was conducted using the methodical framework of Arksey and O'Malley (Arksey and O'Malley 2005), the PRISMA Extension for Scoping Reviews (Tricco et al. 2018), and we began a comprehensive search within biomedical databases using a combination strategy of medical subject heading keywords, terms, phrases, and Boolean operators (see Data S1). The following 14 databases were searched: EBSCOHost; BIOSIS; CINAHL; ScienceDirect; ArticleFirst; Biomed Central; BioOne; ProQuest; SAGE Reference Online; Scopus; SpringerLink; PubMed; Taylor and Francis; and Wiley Online. The search strategies were adapted according to the indexing systems of each respective database (see Data S1).

### 2.3 Study Selection and Data Extraction

**FIGURE 1:**
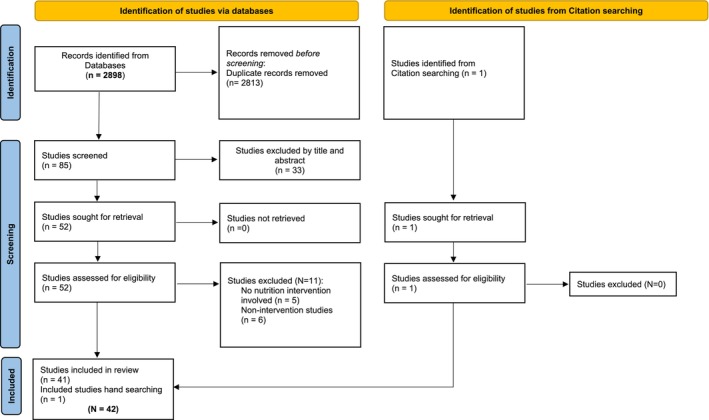
Search flow diagram following PRISMA 2020 Guidelines.

Two of the authors conducted searches for relevant articles, and one author utilized Rayyan QCRI software (Ouzzani et al. 2016) to assist in the screening process. All retrieved articles were screened for relevance to the topic (see Figure 1). In addition, reference lists from retrieved articles were also hand reviewed to identify any additional relevant publications. Titles and abstracts were screened for relevancy, and potentially relevant journal abstracts were reviewed by three of the authors. Potential articles for inclusion in this review were evaluated for relevance, merit, and inclusion/exclusion criteria (see Table 1). Articles accepted for inclusion were individually reviewed by each author. Additionally, the reference list of each included article was screened for potentially eligible articles. Once the list of selected studies was finalized, two of the authors extracted and cross‐checked each study. One author updated the search, reviewed the articles, and wrote the first draft of the results and discussion sections of the review. Disagreement was resolved through consensus, and differences in opinion in data extracted were discussed to reach consensus and tabulated. Given that methodological quality assessment is not a prerequisite for scoping reviews, we did not appraise the included studies (Peters et al. 2020).

## 3 Results

Below are the results for the authors, years published, types of subjects, city/area, major focus related to nutrition intervention in schools, study design, study effects, duration of study, theoretical framework or approach to interventions, and principals undertaking the interventions (see Tables 2 and 3).

**TABLE 2:** Summary findings of eight characteristics in studies reviewed (*N* = 42).

Authors (year)	No. of participants (age; grades)	City/area	Dietary behaviors/knowledge addressed	Intervention duration	Follow‐up period	Theoretical framework/model	Delivered by
CHILDREN							
Educational							
Rosi et al. (2016b)	145 (8–10 yo; 4th grs.)	Parma	Importance of carbohydrates	1 lesson	Self‐ admin. questionnaire after 1 lesson	Health promotion model	Nutrition educator; nut. educator plus humanoid robot
Panunzio et al. (2007)	521 (4th grs.)	Foggia	F&V consumption	36 weeks	Students recorded their daily food and snack consumption throughout the 24‐week intervention and for an additional 12 weeks.	Health promotion model	Teacher‐led vs. nutritionist‐led comparisons made
Watutantrige‐Fernando et al. (2018)	949 (6–10 yo)	Three different geographic areas of the Veneto region	Awareness of importance of iodine, use of iodized salt, and dietary iodine intake	1 year	At the baseline and after 6 mos. students answered questionnaires testing their knowledge about iodine prophylaxis and their eating habits.	Health promotion model	Primary school teachers
Panunzio et al. (2010)	199 (grs. 2–5)	Foggia	F&V intake	15‐weeks with three intervention phases	Three intervention phases	Health promotion model	Teachers after training by nutritionists
Agozzino et al. (2007)	644 (primary school age)	Naples	Overall food intake to measure “correct” eating habits	Pre food intake questionnaire T0; post questionnaire T1; half of school year	6 mos. after educational interventions	Health promotion model	Primary school teachers
Panunzio et al. (2011)	804 (2nd–5th grs.)	Puglia region	Promote F&V intake	15 Week education program	Trial divided into three periods, each consisting of 5 weeks (1st, 2nd, and 3rd intervention periods)	Health promotion model	Teachers trained by nutritionists (Group 1); teachers completed a self‐training course (Group 2).
Roccaldo et al. (2017)	494 (8–10 yo; 4th grs.)	Padova, Rome, and Naples	Adherence to Mediterranean diet	During the 2014–2015 school year	End of school year	Health promotion model	Teachers
Roccaldo et al. (2024)	494 4th–5th graders	North, Center, and South	Adherence to Mediterranean diet	Two school years between 2014 and 2016	End of 1 year after first study	Health promotion model	Teachers
Sotgiu et al. (2009)	353 (6–10 yo)	Cagliari	Develop children's knowledge of Mediterranean diet (MD)	6 mos.; activities happened once a week	At the end of the 6‐month activity timeline. Scouts filled in a questionnaire with images instead of written answers.	Health promotion model	Cub Scout leaders; plus, three adult stakeholders
Vanelli et al. (2014)	632 (9–11 yo)	Parma	Promoting breakfast in primary schoolchildren	3 years	All children enrolled into this study were requested on the 1st summer sport school day to answer a questionnaire on their breakfast habits.	Health promotion model	School, family; pediatricians; sports school, media by different actions
Angelico et al. (1991)	150 (6–7 yo)	Sezze	General Health and Nutrition Edu.; adoption of “prudent diet;” physical activity to a lesser degree	Several lessons taught throughout school year	5‐year follow‐up	Health promotion model	School teachers; involved parents sometimes
Bonaccorsi et al. (2002)	600 (7–8 yo; 2nd grs.)	Firenze	F&V, after dinner snacks, use of vegans, peas, and beans	5 meetings, every 2 weeks	In Weeks: 3, 4, 7–9, and 12	Health promotion model	Primary school teachers
D'Egidio et al. (2021)	76 (6–8 yo; 2nd and 3rd grs.)	Rome	F&V, pasta, legumes, eggs, fish, dietary products, meat, olive oil, sweets, fast‐food, and fruit juice consumption	5 mos. during school year	After 5 mos. of interventions	Health promotion model	Classroom teachers
D'Addesa et al. (2006)	180 (8–9 yo)	Rome	Promote F&V and legume consumption	During school year	After all intervention lessons; end of year	Health promotion model	Teachers and parents
Rosi et al. (2016a)	8165 (8–11 yo.; 3–5th grs.)	Parma	Nutritional knowledge	3 mos., 3‐h classes	Pre‐ and posttest	Health promotion model	Outside experts on nutrition ed.
Pampaloni et al. (2015)	176 (4th and 5th grs.)	Florence	calcium, vitamin D, dairy products, and total caloric energy	7 mos.	Pre and postquestionnaires of students	Health promotion model	Teachers, nutritionists, and parents
Viggiano et al. (2018)	1313 (7–11 yo.)	Campania	Consumption of “healthy food” and decrease in “junk food”	18 mos.	8 and 18 mos.	Health promotion model	Primary school teachers
Laureati et al. (2014)	560 (6–9 yo.)	Milan	Food exposure to food neophobia and liking F&V	6 mos.	At 6 mos. assessment	Health promotion model	Not reported; likely teachers
Trento et al. (2012)	482 (4.5 yo; preschool)	Turin	Promote good food choices and balanced mental and physical development	2009–2011 school years (3 years)	End of this descriptive study	Health promotion model	Teachers
Marconi et al. (2022)	1135 (8–11 yo.; 3rd–5th grs.)	Brescia municipality area	Assess the impact of a nutrition training program for primary school teachers on school children's eating habits.	2 school years	End of study	Health promotion model	Teachers
Scazzocchio et al. (2021)	1235 (8–10 yo; 3rd–5th grs.)	Small town outside of Rome	Increase health and food literacy for F&V	Throughout the school year	Questionnaires were given to students at the beginning and the end of the study.	Health promotion model	Teachers and families
Presti et al. (2015)	672 (5–11 yo)	Acireale, Camporotondo, and San Pietro Clarenza, Sicily	Increase classroom consumption of home‐provided F&V in obese, overweight, and normal‐weight children	16 days	Monitoring throughout the intervention	Health promotion model	Teachers and researchers
Rosi et al. (2015)	76 (8–10 yo; 4th grs.)	Parma and Milano	Improving children's eating habits; intakes of F&V and juices, and dietary total antioxidant capacity	3 mos.	Used 3‐day food diaries before and after intervention	Health promotion model	Classroom teachers
Multicomponent							
Centis et al. (2012)	209 (4th grs.)	Small towns and villages, suburban Bologna	Reinforce the importance of healthy nutrition and regular physical activity	9 mos.	4 mos., then 8 mos. at end	Health promotion model	Univ. faculty in exercise and sports sciences, and parents
Galli et al. (2012)	2,151 (9 yo)	Parma	Eating breakfast daily	During 1 school year	Directly interviewing the children's parents or guardians	Health promotion model	Professionally guided physical exercise and nutrition education in schools
Gallotta et al. (2016)	230 (8–11 yo; 3rd–5th grs.)	Rural area north of Rome	Focused on F&V to encourage good eating habits	5 mos.	Pretest; posttest measures: PA; eating habits, and body fat mass then postintervention	Health promotion model	Generalist teachers after training by nutritionists
Haerens et al. (2009)	155 (2–8 yo.)	8 different countries including Italy	Learn about overall eating habits at home and school	3 mos.	Focus groups of children and parents	Descriptive study	Focus group moderators
Piana et al. (2017)	190 (2nd—5th grs.)	Spoleto, Umbria	Improve personal awareness, promote healthy food choices, and increase levels of physical activity	4 mos.	End of study	Community‐based approach.	Family members, schools, university, families, sports societies, farms, mass media, and municipalities.
Grassi et al. (2016)	60 (10 yo; 5th grs.; and parents)	Treviso	Impact of a school‐based nutrition and media education intervention on the promo. of V&F consumption	10 weeks	Data collection occurred at three points in time: pre‐, post‐ (directly upon completion of the intervention), and delayed posttest (3 mos. after completion of the intervention).	Health promotion model and media literacy	Dietician and media educator
Scuderi et al. (2016)	1000 (6–11 yo; 1st, 3rd, and 5th grs.)	Italy (unspecified)	Increase the familiarity with a variety of F&V to increase consumption and awareness of these as part of a healthy diet	1 school year	Baseline measurements followed by measurements after yearly intervention using a questionnaire.	Health promotion model	Classroom teachers
Nosi et al. (2021)	863 (3rd and 4th grs.)	4 cities in Italy, 2 North, 1 South, 1 central	Healthy nutrition and lifestyle	1 School year	Pre–Posttest beginning and end of school year	Social marketing model	Tutors, teachers, parents, and community
ADOLESCENTS AND CHILDREN							
Educational							
Ceratti et al. (1990)	12,354, (3—18 yo.)	Milan	Proper diet education and risks of being overweight	2 school years	5 and 12 mos.	Health promotion model	School doctors and health assistants
Viggiano et al. (2015)	3110 (9–19 yo.)	Campania	Promote nutrition edu. and improve dietary habits	6 mos.	6 and 18 mos.	Health promotion model	School teachers
Multicomponent							
Tripodi et al. (2011)	10, 000 (3–14 yo; & 100,000 adults, in community)	Modena	Promoted healthy nutrition and regular physical activity in schools and local communities	From 2004 to 2011 (publication of study)	Unspecified but ongoing	Social cognitive theory	School teachers
Mazzeschi et al. (2014)	90 (5–10 yo); 90 (11–16 yo) with overweight or obesity	Umbria	Multidisciplinary approach to improving three key aspects of healthy lifestyles: nutrition, exercise, and psychological aspects with the strategy of a family‐based approach	4 years	(1) Anthropometric measurements performed by family pediatricians every 5 years; (2) a survey “OKKIO alla Salute” of a significant number of families from Umbria every 2 years; and (3) a survey Studio PASSI of a significant number of families from Umbria every 2 years	Community and school‐based multidimensional intervention program	Pediatricians, teachers, parents, and other community members, media outlets
Franceschi et al. (2021)	30 (primary school classrooms; 96 obese students participating in the clinic program)	3 different school districts in provinces of Bolzano, Trento, and Verona	An app and a cookbook promoting transcultural nutrition and a healthy lifestyle; no‐cost physical activities organized. Healthy lifestyle teaching	6 mos.	From November 2018 to April 2020, in the three obesity clinics; 97 patients accepted enrollment in the project	Integrated and comprehensive education and clinical care model	Teachers, parents, and pediatricians
ADOLESCENTS							
Educational							
Amaro et al. (2006)	241 (11—14 yo)	Naples	Test the efficacy of the game Kalèdo on changes in nutrition knowledge and dietary behavior	24 weeks	Pre‐ and post–follow‐up	Health promotion model	Classroom teachers
Varì et al. (2022)	566 (11–13 yo)	Towns outside of Veneto, Lazio, and Basilicata	Increase food literacy for digestive process, nutrient functions, and nutrient recognition	Throughout the school year	3 questionnaires given to students at the beginning and at the end of the study	Health promotion model	Expert nutritionists, teachers, and families
Lazzeri et al. (2013)	4218 (13–15 yo)	5 regions in Italy: Tuscany, Apulia, Campania, Marche, and Sicily	Increase F&V level in school	3 mos.	T‐1 beginning and T‐2 end of study	Health promotion model	Specially trained teachers and local health unit personnel
Fraticelli et al. (2016)	65 (17–21 yo)	Chieti	Healthy nutrition in adolescents	3 weeks	T0, T1, T2; all 1 week apart	Health promotion model	Research members
Multicomponent							
Latino et al. (2023)	100 (14–15 yo; high school)	Southern Italy	Nutrition edu. program, unspecified	12‐week classroom‐based physical activity break program	After the 12‐week intervention	Health promotion model	High school teachers
Ermetici et al. (2016)	487 (11–15 yo; middle school)	Townships in an urban area around Milan	Evaluate whether a school‐based multicomponent educational program could improve adiposity measures in middle school adolescents	2‐school year intervention	Anthropometric measurements for all students in both groups and a questionnaire were completed at the beginning and the end of the 1st and the 2nd school year.	Health promotion model and an environmental control	Teachers, parents, outside experts

Abbreviations: edu.: education; F&V: fruits and vegetables; grs.: grades; mos.: months; NR: not reported in the study; yo: year old.

**TABLE 3:** Study design, overall study quality, and overall intervention effectiveness on anthropometrics and dietary behavior of reviewed studies (*N* = 42).

Authors * (year)	Study design	Study effect on anthropometrics	Study effect on dietary knowledge and/or behavior and instrument used to measure	Study effect on other assessed health attitudes, behaviors, outcomes
CHILDREN				
Educational				
Rosi et al. (2016b)	Group RCT pilot using self‐administered qtns. on nutrl. knowledge	NA	Self admin qtn. showed significant increase in nutrl. knowledge of children involved in nutr. educator led (58) and educator‐led plus a game‐based robot (54), single‐lesson, edul. Intervention. Presence of a humanoid robot in teaching activity did not result in any significant learning improvement. Two intervention groups outperformed control group (33) in a cultural–nutrl. awareness factor (AF) score.	NA
Panunzio et al. (2007)	RCT evaluated the effectiveness of a school‐based intervention by the classroom teacher compared to a nutritionist‐based intervention, with the primary endpoint an increase in the children's consumption of F&V and legumes and a decrease in consumption of chips and sugar‐sweetened drinks.	NA	Using food consumption measures, this study implies that nutrition education and application intervention by teachers may promote F&V and legume consumption in elementary school age children, as well as if not better in some cases than nutritionist‐led interventions.	NA
Watutantrige‐Fernando et al. (2018)	Group RCT with students using a self‐administered qtn. on knowledge of iodine prophylaxis and use of iodized salt	Urine samples were collected from a randomly selected subgroup of participants (313 at T0 and 312 at T1) 6 mos. The median UIC rose significantly in this group, from 70 μg/L (minimum 30, maximum 361, SD 70.95) at T0 to 91 μg/L (min 18, max 392, SD 83.7) at T1 (*p* < 0.05). This applied to both the Italian‐origin SAC and the foreign‐origin SAC. The UIC did not significantly differ between T0 and T1 in the boys’ samples, whereas the girls’ UIC rose significantly from T0 to T1 in both the girls of Italian origin and those of foreign origin. Milk and iodized salt intakes were associated with better iodine status per se, and more so when used simultaneously.	Edul. intervention improved the children's knowledge via qtns about iodine prophylaxis and use of iodized salt. From T0 to T1 there was a significant improvement in respondents' knowledge about iodine prophylaxis (from 44% to 70%), iodized salt consumption (from 78% to 84%), and median urine iodine concentrations (from 70 to 91 μg/L). Milk and iodized salt intakes were associated with a better iodine status per se, and more so when used simultaneously. Girls drank milk less often than boys did (daily in 52% and 59% of cases, respectively). Children of foreign origin ate sodium‐rich food more often than Italians did.	NA
Panunzio et al. (2010)	Student diaries measured F&V intake during three phases: 1–5, 6–10, and 11–15 weeks, during a 15‐week nutrl. edu. program called to promote F&V. In addition, F&V was monitored in a subsequent follow‐up period from the 16th to the 32nd week.	NA	A significant increase in fruit intake (≥ 2 p/d) occurred in 92 (*n* = 99, 46.2%, *p* < 0.001) and in vegetable intake (≥ 2 p/d) in 91 (*n* = 99, 45.7%, *p* < 0.001) schoolchildren respectively via food intake diaries. The schoolchildren reported a constant increase in F&V consumption during the 1st to the 10th week and maintained this enhanced consumption frequency during the third phase (11th–15th week), and during the follow‐up (from the 16th–32nd week).	
Agozzino et al. (2007)	Administered a frequency of food intake qtn., before and after the intervention (6 mos.). To compare the answers given before and after the edul. intervention the Wilcoxon test was applied to dependent data	NA	Study confirmed that a substantial percentage of children do not show a proper nutr. intake and therefore nutr. edu. interventions are opportune and necessary.	NA
Panunzio et al. (2011)	RCT comparing teacher effectiveness of promoting F&V among students with one group of teachers trained by a nutritionist and the other taking a self‐training course; child participants completed a daily dietary diary recording the food they ate the day before.	NA	F&V. consumption increased significantly in the students of Group 1 (*n* = 409), whose teachers attended a course held by nutritionists., in 183 (44.7%) and 157 (38.3%) students, respectively; Group 2 (*n* = 395) whose teachers completed a self‐training course recorded an increase in F&V consumption in 81 (20.5%) and 76 (19.2%) students, respectively.	NA
Roccaldo et al. (2017)	Pre–posttest intervention design of children's adherence to the MD was assessed by the KIDMED test. Children were asked how many times a day they ate fruit. Height was measured to the nearest 0.1 cm by a stadiometer SECA 214TM. Body weight was recorded to the nearest 50 g using an electronic scale SECA 872TM. A simplified procedure to measure body weight was used to encourage children and their parents to take part in the study.	Not conducted for changes. Measure of height and weight was done to encourage parent/student participation.	Intervention group, the proportion of children with high adherence to the MD significantly increased in the total sample, females and South in the postintervention; no significant changes in levels of adherence related to ponderal status were detected. A significant increase was found, particularly in the proportion of children who improved their frequency of consumption of F&V daily, especially in the South.	Italian Fruit School Scheme getting free F&V
Roccaldo et al. (2024)	Control Trial with Pre–posttest intervention design of children's adherence to the MD was assessed by the KIDMED test	Not conducted for changes. Measure of height and weight was done to encourage parent/student participation. However, students in ponderal status classes were tracked.	Improvements in the high and low adherence rates to MD were observed. The % of subjects with optimal adherence improved in both sexes, in all the geographical areas, and in ponderal status classes	Italian Fruit School Scheme getting free F&V
Sotgiu et al. (2009)	A mix of stakeholder adults involved the Cub Scouts in games, to teach the characteristics of foods. They promoted knowledge of, and experimentation with, healthy foods that are less familiar to the children.	NA	Qtns. and reports showed improvements in the Cub Scouts' knowledge and interest in nutr.	NA
Vanelli et al. (2014)	Two groups of children were interviewed by a multiple choice qtn. on their breakfast habits. Group 1 counted only the children who underwent the intensive campaign (*n* = 341), and Group 2 included those matched peers who did not attend any breakfast‐promoting program (*n* = 291).	Height was measured. Body weight was measured in minimal clothes on portable and calibrated scales. Body mass index (BMI) was calculated using the formula: weight (kg)/height (m^2^).	Children who did not eat breakfast were found to be more numerous in Group 2, who did not attend the program (17.5%) than in Group 1 (8.0%; *p* = 0.0001) who did not attend. An intensive breakfast‐centered strategy seems to be effective in breakfast promotion and overweight risk decrease. At the end of a 3‐year intervention in Parma schools and families, a significant reduction in breakfast omission was observed in children who participated in the present breakfast promotion‐based study compared with children who did not	NA
Angelico et al. (1991)	Four consecutive annual cross‐sectional surveys of students. Weekly composition of school snacks recorded by children on an *ad hoc* diary at the beginning of the study and 5 years later. The results were checked daily by teachers.	Height and weight; WHO BMI calculated; control of hypercholesterolemia, high blood pressure, and obesity. The conclusion was that school‐delivered programs of general nutr. edu. for the control of risk factors does not appear to be able to control child obesity.	Voluntary changes in the habitual diet, using four consecutive annual cross‐sectional surveys of students.	NA
Bonaccorsi et al. (2002)	Epidemiological investigation of food habits observed pre/post edul. intervention	NA	Increased consumption of fresh fruits (40%), decreased consumption of after‐dinner snacks (3.2%), and increased consumption of vegetables, peas, and beans (13.2%)	Several meetings were organized over the intervention period to involve the parents of the students.
D'Egidio et al. (2021)	Single‐arm field trial. 2nd‐ and 3rd‐grade classes of primary school attended an oral presentation about nutr. and physical activity, thereafter, involved in three game sessions for 5 months	NA	Two multiple‐choice qtns. were administered at the beginning and the end of the trial. Overall, the study showed that oral presentation combined with game sessions was effective in improving children's self‐reported dietary habits and physical activity behavior.	NA
D'Addesa et al. (2006)	Parents invited to attend three meetings on neutral. and sensory matters. All teachers were trained on neutral. and sensory aspects of food, as well as motivational and psychological factors affecting choices.	NA	Before intervention on children, dietary habits, particularly those related to the target food items were investigated by qtns. Results showed a better trend of F&V and legume consumption, an increased percentage of children having breakfast every day and consuming fruit at each meal. The percentage of subjects eating legumes and vegetables at lunch and dinner also increased. According to the preliminary data obtained, we can hypothesize that NEI model we experienced with the involvement of school and family may promote healthy dietary patterns.	NA
Rosi et al. (2016a)	“Learning through playing” approach. Students taught key concepts of nutrl. themes; later, the Master of Taste and children played edul. games, aiming to increase children's understanding.	NA	Qtns. were analyzed. Children nutrl. knowledge significantly increased (*p* < 0.001) in all school grades.	NA
Pampaloni et al. (2015)	Project messages and materials featured Mister Boner, a bony little boy, who is the hero of a cartoon game series and several online games, all produced to attract children to the project and to involve the entire family. The neutral. edu. program had a dedicated website with the edul. program, “online” games, and scientific material on bone health; a “Mister Bone” Calendar through which each child could check their physical activity and daily calcium intake; A classroom session conducted by nutritionists to teach children the importance of adequate intake of calcium and other nutrients necessary for healthy bones. A “student kit”	NA	A significant increase in calcium (from 870 ± 190 to 1100 ± 200 mg/day, *p* < 0.05), and vitamin D (from 3.6 ± 1.53 to 4.1 ± 2 g/day) intake in children was documented after the edul. program. The amount of specific foods important for bone health consumed, such as milk and vegetables, increased significantly, both in male and female children (*p* < 0.05). There was no significant change in the total caloric intake.	NA
Viggiano et al. (2018)	RCT with two groups: (1) the treatment group consisting of playing the board game Kaledo over 20 sessions and (2) the no‐intervention group.	Teachers helped students complete the WHO Health Behavior in School‐Aged Children (HBSC) physical activity qtn. BMI *z*‐score was significantly lower in the treated group compared to the control group at 8 months. Frequency and duration of self‐reported physical activity were significantly augmented in the treated group at both postassessments.	Students completed a 1‐week food diary at each assessment (pre‐ and postintervention). Significant increase in the consumption of healthy food and a significant decrease in junk food intake was observed in the treated group.	NA
Laureati et al. (2014)	An intervention and control group to see whether and how the application of the “Food Dudes” multicomponent school‐based intervention, consisting of rewards, peer‐modeling, and repeated exposure to F&V, influenced the liking of such food, in addition to food neophobia.	NA	Main were that the “Food Dudes” dietary intervention program is effective in reducing food neophobia and, most importantly, that this effect is also observed over the long term (6 months). Additionally, the program was successful in increasing the liking for F&V, although the effect was more pronounced for fruit.	NA
Trento et al. (2012)	33 games and stories aimed at helping children understand the value of food and healthy eating. The project was included in the curricula of six schools in the form of edul. activities, workshops, and open classes.	NA	Four schools favored the psychomotor skills and social mobility areas, and three opted more for visual–spatial and visual–perceptual abilities. Four schools also promoted the area of language to foster better understanding of food and healthy eating.	NA
Marconi et al. (2022)	From participating schools, 11 volunteer teachers attended a nutr. training program NTP (trained teachers; TT), and as a control, authors identified a group of teachers (untrained teachers; UT) who did not attend the NTP. The effect of NTP intervention was evaluated on 599 children in the TT group and 536 in the UT group, respectively, using the KidMed qtn. to assess adherence to an MD.	NA	To evaluate children's eating habits, we estimated adherence to an MD using the KidMed qtn. This is self‐administered and focuses on specific food habits characteristic of the MD. The scores were calculated from responses to 16 yes/no questions concerning the consumption of F&V, fish, legumes, pasta and rice, cereals, grains, olive oil, yogurt, cheese, and sweets. Some specific questions about breakfast and fast‐food habits were included. Compared with the UT group, children in the TT group had significantly higher adherence to an MD (Wilcoxon test *p* = 0.012) and showed significantly healthier habits regarding fast‐food consumption, the eating of sweets, and breakfast composition.	Used various games to promote the psychophysical development of the child which in turn promotes healthy eating
Scazzocchio et al. (2021)	A didactic PowerPoint presentations on “Seeds & Fruits” and “Food Chains,” and four experimental and practical activities aimed at increasing knowledge and familiarity with vegetables. Some recipes on seasonable vegetables were provided to induce the students to experiment with new tastes.	NA	Results showed a significant increase in right answers at T1 with respect to T0 (*z* = 2.142, *p* = 0.032). Fisher's exact probability test showed an answer variability depending on the issue considered. In conclusion, this work could be considered as a first necessary step toward the definition of new edul. program, aimed at increasing food literacy and favoring a healthier relationship with food, applicable in a widespread and effective manner.	NA
Presti et al. (2015)	*Food Dudes Healthy Eating Program* is a behavior change program based on three core principles: (1) role modeling, 2) rewards, and (3) repeated tasting. For 2 consecutive days, in both intervention and comparison schools, trained researchers assessed all food that was brought from home for the midmorning snack. Thereafter several phases of F&V instruction were given, and home learning packets were given while student F&V intake was monitored, with rewards given for healthy choices at snack time.	Body mass index scores were calculated using children's weight and height, at the beginning of the study. Children were ranked as underweight, normal, overweight, or obese according to International Obesity Taskforce criteria. This was done for comparison groups only. No follow‐up weights were taken.	Main outcome measures were grams of F&V brought from home and eaten. Intervention schools show a significant increase in home‐provided F (*p* < 0.001) and V (*p* < 0.001) consumption both in overweight and nonoverweight children. Approximately half of children in the intervention schools ate at least one portion of F&V at the end of the intervention and maintenance phases.	NA
Rosi et al. (2015)	A single‐group edu. intervention involved a 3‐month nutrl. program including lessons and edul. videogames. A technology platform for games and edu. (edutainment) was developed to teach children healthy eating and lifestyle habits through games	NA	Intakes of F&V, juices, and dietary total antioxidant capacity (TAC) were measured using 3‐day food diaries before and after the intervention. The daily total consumption of F&V increased from 421.8 (320.3) to 484.3 (337.2) g/day (*p* = 0.016). Consequently, daily dietary TAC increased by 26%, rising from 1.4 (1.3) to 1.6 (1.3) mmol of Trolox equivalents (*p* = 0.006).	NA
Multicomponent				
Centis et al. (2012)	Parents complete a qtn. on habitual family behavior; measured children's anthropometric parameters and recorded their habits in terms of physical activity. In the intervention group, parents and teachers received more intense lifestyle counseling, associated with weekly motivational telephone calls to families to motivate further their lifestyle changes.	A multicomponent intervention in a school district, proposed as playful activity to children, addressing the needs of teachers and empowering parents, was able to modify the time trend of progressive increase in body mass index (BMI), compared to data obtained in a control group. Reduced standard deviation score BMI was accompanied by changes in behavior activity, in the time spent in outdoor games and physical activity.	NA	Means of transportation; limit television viewing; movement and active play
Galli et al. (2012)	Data on the number of sports practiced (one or more), physical activity level (as minutes of physical activity per week), and breakfast eating (yes/no) were obtained by directly interviewing the children's parents or guardians	Anthropometric measurements focused on BMI and fat mass. Taken together, our data show that fat mass can be used to accurately evaluate physical activity and eating habits in children, and suggest that, in preventive health programs, the fundamental parameter to pay attention to is the amount rather than the type of physical activity.	Breakfast skipping was significantly correlated with percent fat mass only in underweight girls.	Open‐air games and TV watching were secondary outcomes.
Gallotta et al. (2016)	Evaluate the efficacy of three 5‐month combined PE and nutr. edu. on body composition, (PA) level, sedentary time, and eating habits of schoolchildren; in a cluster‐randomized controlled intervention; three groups	Body fat mass % increased after intervention in all groups: experimental Group 1 vs. experimental Group 2 vs. control group	Children significantly changed their consumption of some specific foods after the intervention period (potatoes, meat, cold cuts, eggs, sweets, dairy products, and snacks). Eating F&V increased.	NA
Haerens et al. (2009)	Twenty focus groups with children (74 boys, 81 girls) and 36 focus groups with 189 parents (28 men, 161 women) were conducted.	NA	Only in 2 of 8 countries did children mention receiving nutr. edu. at school. Rules at home and school ranged from not allowing the consumption of unhealthy products to allowing everything. Parents mentioned personal (lack of time, financial constraints, preferences), socio‐environmental (family, peer influences), institutional (school policies), and physical–environmental (availability of unhealthy products, price, season) barriers to healthy eating.	Sedentary time significantly decreased in children of all groups.
Piana et al. (2017)	Interventions addressed healthy food choices, lifestyle, and physical activities and were structured in four phases over 4 months. The KidMed test (a validated index based on principles sustaining Mediterranean dietary patterns as well as those that undermine them) and open‐ended qtns. (to highlight the key components that contributed most to the beneficial effects) were used to assess the effectiveness of the intervention. KidMed scores were evaluated both before and after intervention (T0–T1) and the written answers collected (from teachers, parents, and children) were subjected to content analysis using a form of grounded theory.	NA	Data points to a significant before/after increase in KidMed scores (*t* = −3.88; *p* = 0.000), revealing an increase in children's adherence to the MD, healthy habit changes, and greater parental awareness of their edul. responsibilities regarding food choices as well as physical activity.	A new school‐family alliance was a result of the edul. intervention.
Grassi et al. (2016)	10‐week‐long intervention included sessions on nutr. edu. and media literacy. Included a health communication media‐based campaign workshop during which the children created posters, newsletters, and video commercials related to fruits and vegetables targeted to their parents. Quasi‐experimental study (with one intervention group and one control group) and a focus group study.	NA	Evaluation used a mixed‐methods approach, including a quasi‐experimental study (with one intervention group and one control group) and a focus group study. Children completed self‐administered surveys, which included items and scales measuring F&V consumption, motivation, self‐efficacy, and perceived parental social support related to F&V consumption. Data indicate that this intervention was effective for children but not for parents. Evaluation results show that the intervention was effective in significantly increasing children's F&V intake (*p* < 0·05) and all psychosocial determinants (*p* < 0·05).	NA
Scuderi et al. (2016)	The tools provided by the program to disseminate the identified messages are free distribution of F&V; information campaign on the characteristics of the F&V products in terms of quality, nutrl. value and health aspects, seasonality, territoriality, and respect for the environment, addressed both to teachers and parents, in order to prolong the effect of induction on the consumer; use of suitable equipment for the utilization and tasting of the products distributed; initiate and consolidate the creation of a network of scholastic institutions willing to participate on an ongoing basis to the program	NA	Changes in attitudes toward self‐efficacy and willingness to eat F&V varied by grade level. For example, posttest, third‐grade students reported more positive attitudes toward eating F&V (*p* = 0.02), the belief that they could eat more F (*p* = 0.05), and the willingness to try new F (*p* = 0.25), but this pattern was not evident among fifth‐grade students. Among first‐grade students, willingness to try new F (*p* = 0.52), willingness to try new V (*p* = 0.09), and beliefs that they could eat more V (*p* = 0.21), decreased significantly.	NA
Nosi et al. (2021)	Social marketing campaign organized around the four Ps of the marketing mix (*product*: the educational activities; *place*: the involved schools and supermarkets; *promotion*: the in‐person and technology‐based communication; and *price*: hours spent by the targeted children in fulfilling the educational activities.	Reduced their sedentary behavior	Findings suggest that social marketing education campaigns can be effective tools to improve children's knowledge about healthy food and lifestyle, reduce their sedentary behavior, and increase their consumption of healthy food. Also, increasing children's acceptance of healthful nourishment is a valuable tool to improve the dietary habits of the entire family.	NA
ADOLESCENTS AND CHILDREN				
Educational				
Ceratti et al. (1990)	Doctors gave information to students and their parents on risk of being overweight and obese. Students were asked to keep weekly food consumption diaries. (Parents for younger students.)	Intervention reduced % weight excess (from 33.6+/–0.5% to 28.8+/–0.5% after 12 months, *p* < 0.001); 67% of obese subjects lost weight, and body weight returned within normal limits in 31% of subjects.	An edul. didactic strategy was used but changes in knowledge were not assessed post‐ and preintervention.	NA
Viggiano et al. (2015)	A two‐group design (treatment and control) with one pretreatment assessment and two posttreatment assessments was employed. Intervention was students playing the board game Kaledo played each week for 20 consecutive weeks	The treated group had significantly lower BMI *z*‐score with respect to the controls at 6 mons. and at the second (18 mons.) assessment.	The treated group obtained significantly higher scores than the control group on the Adolescent Food Habits Check List qtn. on four sections of the dietary qtn. nutr. knowledge, healthy and unhealthy diet and food, food, and physical activity.	NA
Multicomponent				
Tripodi et al. (2011)	Interventions included teacher training, physical activity promotion, nutr. edu., printed material distribution among pupils and families, and modification of school meals.	NA	Initially, assessed the children and their families’ behaviors and attitudes as well as their nutrl status. Some improvements in F&V daily intake were made during school meals.	They are establishing alliances with sports clubs to promote sports and physical activity both in school and outside school (after school) for children and adolescents. They organized hike excursions for children and their families.
Mazzeschi et al. (2014)	Coordinated, capacity‐building approach that aims to reduce childhood obesity through a social process in which local environment, childhood settings, and family norms are directed and encouraged to facilitate the adoption of a healthy lifestyle in children	Article explains the specific aims and protocol of the program. Initiative not started at the time of the publication.	Article explains the specific aims and protocol of the program. Initiative not started at the time of the publication.	Article explains the specific aims and protocol of the program. Initiative not started at the time of the publication.
Franceschi et al. (2021)	An app and a cookbook promoting transcultural nutr., and a healthy lifestyle were developed, and no‐cost physical activities were organized. Healthy lifestyle teaching was implemented in 30 primary school classrooms.	At the obesity clinic, the BMI *z*‐score was not significantly decreased.	Learning was assessed through pre‐ and postintervention qtns. Primary school students increased their knowledge about healthy nutr. and the importance of physical activity (*p* < 0.001). At the Obesity Pediatric Clinic, after 6 months, pre–post‐intervention variation in their consumption of F&V was +14% (*p* < 0.0001) and no variation in physical activity habits occurred (*p* = 0.34).	NA
ADOLESCENTS				
Educational				
Amaro et al. (2006)	A simple two‐group design (treatment and control) with pre‐ and postassessment. RCT intervention group engaged in 15‐ to 30‐min‐long play sessions once a week	Qtn. on physical activity given. Anthropometric measurements were carried out. Control group did not engage in physical activity sessions. No significant difference between treated group and control group at postassessment controlling for baseline values in BMI was found	A qtn. was given to the participants at the beginning and at the end of the study to evaluate nutr. knowledge, physical activity, and food intake. Children playing the game Kalèdo showed a significant increase in nutr. knowledge (*p* < 0.05) and in weekly vegetable intake.	NA
Varì et al. (2022)	The CO group underwent a “traditional” nutr. edu. program, attending curricular science lessons and one lesson (2 h) focused on the basic nutr. principles of a balanced diet as indicated by the Food Pyramid, held by an expert nutritionist. The MaestraNatura (MN) group participated in all the learning activities provided by the MNP edul. path “We Are What We Eat” which included three didactic PPT presentations and food recipes to take home.	NA	The results showed a significant improvement in knowledge (*p* < 0.001) in the MN group with respect to the CO group for all the qtns. Furthermore, students' ability to transfer the principles of nutr. guidelines to the real context of daily meals was determined by asking the MN group to create a weekly food plan before (T0) and after (T1) the completion of the MN program. MN group demonstrated improved performance in organizing the weekly menu plan at T1 with respect to T0 (*p* = 0.005).	NA
Lazzeri et al. (2013)	Randomly selected subjects from middle and high schools in five Italian regions; subjects were randomly divided into two groups: intervention (I) and control (C). A qtn. on fruit and vegetable consumption was administered, at the baseline as well as at the end of the period with the intervention to determine increased consumption of F&V and Knowledge and Attitudes related to F&V. Middle school intervention used edul. packages for students and families, while the intervention in the high schools was the use of edul. packages and fresh FV offered in the vending machines for students and teachers.	Data were obtained from a total sample of 3408 students on their anthropometric characteristics (age, weight, height, and BMI) of the two groups (middle and high school) for the two arms (I and C). No difference is highlighted, and the two groups appear to be homogeneous with respect to these variables considered.	Results confirmed that adolescent students at baseline survey eat less than the number of F&V consumption recommended by the International Guidelines. After the intervention time, they observed an increase in F&V consumption both in middle and high school students. There was a positive change in the behavior only in the intervention group, given the variance in consumption of F&V in both groups. No major change was seen in the knowledge, attitudes, and responses to the self‐administered qtn.	NA
Fraticelli et al. (2016)	Adolescents were engaged in three supervised group sessions. Measures of healthy food knowledge and game enjoyment were collected during three stages of assessment.	NA	After playing Gustavo in Gnam's Planet, participants significantly improved their knowledge of a healthy diet, compared to the recreational web games; whereas the level of fun experienced while playing the recreational and the educational games was not significantly different.	NA
Multicomponent				
Latino et al. (2023)	Controlled study to analyze the mediating role of a school‐based physical activity program (MVPA) on overweight adolescents' self‐efficacy and academic achievement. Students participated either in a 12‐week classroom‐based physical activity break program performed during science classes (600/2 days per week) in which a nutrl. edu. program was carried out or in regular science lessons (600/2 days per week).	At the beginning and end of the intervention programs, a set of standardized motor evaluation tests (standing long jump test, Harvard step test, push up, and sit and reach test) was conducted. Using a two‐factor repeated measures ANOVA, they found a significant “Time _ Group” interaction for all four Motor tests carried out; but no change in the control group. A meaningful “Time Group” interaction was also reached for BMI (F1,98 = 59.30, *p* < 0.001, *h*2 *p* = 0.37, large effect size). The control group did not report any significant changes (*p* > 0.05).	NA	The scholastic self‐efficacy test and the Amos 8–15 were administered. As a result, a meaningful Time Group interaction for the self‐efficacy variable and Amos 8–15 was observed in the intervention group. Specifically, they reported significant improvement in study skills, motivational factors, concentration, and self‐efficacy, as well as a decrease in anxiety and BMI (*p* < 0.001).
Ermetici et al. (2016)	Nonrandomized control pilot using multicomponent interventions: changes in the school environment (alternative healthy vending machines, and edul. posters) and individual reinforcement tools (school lessons, textbook, text messages, pedometers, and reusable water bottles)	The intervention was associated with a significant difference in BMI *z*‐score (20.1860.03, *p* < 0.01) and WHtR (20.0460.002, *p* < 0.001), after controlling for baseline covariates. Subgroup analysis showed the maximum association between the intervention and the difference in BMI *z*‐score for girls with overweight/obesity.	Physical activity increased and consumption of sugar‐sweetened beverages and high‐energy snacks decreased in adolescents after the intervention.	NA

Abbreviations: edu.: education; edul.: educational; F&V: fruits and vegetables; MD: Mediterranean diet; mos.: months; NA: not applicable; nutr.: nutrient; nutrl.: nutritional; qtn.: questionnaire; qtns: questionnaires; RCT: random control trial.

### 3.1 Timeline

This scoping review involved close to a quarter century of research published about school‐based nutrition education interventions (1990 to December 2024), thus providing a robust look at the nature and extent of this important area of child and adolescent health promotion undertaken across Italy. There were 42 studies published in peer‐reviewed journals during this time. Published studies have been spread consistently throughout this recent decade, except for only two works published in the 1990s (Angelico et al. 1991; Ceratti et al. 1990) and one published in 2024 (Roccaldo et al. 2024). For productivity, the average published per year is 1.75. However, not accounting for the lean years of the 1990s and 2024, productivity increased to 3.5 articles per year.

### 3.2 Subjects

The number of subjects in these studies ranged from 65 to well over 1000 students in those projects that had a population‐based approach to educational interventions. Naturally, projects that had a narrow focus or involved more complex metrics, such as taking body mass index measures or iodine levels, had a smaller number of subjects recruited.

For this review, the types of subjects studied were classified as student children (1–11 years old), adolescents (12 years old and above), or both children and adolescents. Children only were the most studied, involved in 31 (Agozzino et al. 2007; Angelico et al. 1991; Bonaccorsi et al. 2002; Centis et al. 2012; D'Addesa et al. 2006; D'Egidio et al. 2021; Galli et al. 2012; Gallotta et al. 2016; Grassi et al. 2016; Haerens et al. 2009; Laureati et al. 2014; Marconi et al. 2022; Nosi et al. 2021; Pampaloni et al. 2015; Panunzio et al. 2007; Panunzio et al. 2010; Panunzio et al. 2011; Piana et al. 2017; Presti et al. 2015; Roccaldo et al. 2017, 2024; Rosi et al. 2015; Rosi et al. 2016a; Rosi et al. 2016b; Scazzocchio et al. 2021; Scuderi et al. 2016; Sotgiu et al. 2009; Trento et al. 2012; Vanelli et al. 2014; Viggiano et al. 2018; Watutantrige‐Fernando et al. 2018) of the 42 studies, while six studies (Amaro et al. 2006; Ermetici et al. 2016; Fraticelli et al. 2016; Latino et al. 2023; Lazzeri et al. 2013; Varì et al. 2022) involved adolescents alone and five studies (Ceratti et al. 1990; Franceschi et al. 2021; Mazzeschi et al. 2014; Tripodi et al. 2011; Viggiano et al. 2015) involved both youth groups. The grades selected for research in children were spread among primary, elementary, and middle schools. While the rationale given by researchers in this area notes the importance of establishing healthy eating habits and some type of physical activity early in life, this review shows that there is less interest in evaluating school‐based nutrition education in high schools. Opportunities for more insight into working with adolescents are evident, especially knowing the success environmentally focused interventions can have on youth behaviors in other areas of unhealthy consumption such as tobacco and alcohol. As noted above, only six of the 42 studies involved adolescents alone, with five studies having a mix of both children and adolescents.

All studies naturally involved the parents of the youth subjects, given informed consent and IRB approvals required for the former group and needed for most studies in the latter group. Many studies gave detailed briefings of the purpose of the study, over and above the informed consent process, to boost compliance of their children or have them complete activities in the intervention. Some studies have expanded the involvement of the parents as part of the intervention, such as preparing food for the student subjects, serving as purveyors of healthy food messaging, learning activities, or participating in physical activities or family activities. Researchers seem to have had much success in working with school administrators, teachers, and other school personnel.

### 3.3 Sites Selected (City/Region)

This scoping review accounted for the city/region of schools and students recruited for studies. It is fair to say that the schools selected are situated throughout the country. There were 15 studies in the northern region, 11 studies in the southern region, and 10 studies in the central region. There were five studies conducted in two or more regions, and one study did not specify the region of Italy. Efforts were made to involve both the city and rural areas. Little was noted about the socioeconomic status of the residents of the area or areas that were “underserved” in healthcare. A few studies looked at variances in native versus non‐native‐born Italian children.

### 3.4 Dietary Knowledge/Attitudes/Behavior Addressed

The background, rationale, and purpose of undertaking these studies were consistent among all studies. The overall impact sought for nutritional intervention in youth was to primarily engender a “healthy lifestyle” early on that included healthy food choices and ample physical activity. Secondly, the rationale was to avoid or reduce overweight or obesity in children. Therefore, general outcomes addressed (1) increasing knowledge of students about healthy living; (2) changing or forming positive attitudes toward health‐enhancing food choices and physical activity; and (3) changing student (and at times parent) behaviors.

More specifically, the most popular dietary subject addressed was consumption of fruits and vegetables (F&V), followed by legumes. Some studies made specific mention of a “Mediterranean Diet” (Roccaldo et al. 2024; Sotgiu et al. 2009). While a significant number of studies involved students keeping food consumption diaries, avoiding junk food or sugary drinks surprisingly were not prominent outcome measures. Breakfast and lunch meals were a larger focus than dinner and snacks. About 14% of the studies combined a significant focus on physical activity with a dietary message. A few unique areas of focus were the importance of iodine, use of iodized salt, and dietary iodine intake (Watutantrige‐Fernando et al. 2018); calcium and vitamin D intake (Pampaloni et al. 2015); food neophobia (Laureati et al. 2014); and improve adiposity measures in middle school adolescents (Ermetici et al. 2016).

### 3.5 Duration and Follow‐Up Period

The range of duration for the studies was wide, from as little as one lesson to a full school year. There does not seem to be a pattern of duration for studies, other than the quarters of time in a year. Duration is likely related to the study design and time parameters of student, teacher, or researcher availability, rather than knowledge of how long an intervention should be for results. The majority of follow‐ups were pretest/posttest designs. More frequent follow‐ups naturally followed the longer duration of the studies and complexity of outcome measures. Roccaldo et al. (2024) conducted their project over 2 school years (2014–2016).

### 3.6 Theoretical Framework or Model Used for Intervention

First, as a general approach to intervention, 73% of the studies focused on an educational approach, with the balance of 27% using a multicomponent approach. No study focused solely on an environmental approach, and only a few environmental approaches were evident in the multicomponent approaches. Sixty‐two percent (62%) of the studies used a health promotion model. Theory‐driven research is not evident in these studies, given only one study noted a theoretical model, social cognitive model (Tripodi et al. 2011). Three studies used a social marketing or health communication model; one was a descriptive study (Haerens et al. 2009), two used a community‐based approach, and one had a clinical care component (Franceschi et al. 2021).

### 3.7 Intervention Delivered by

About 46% of the studies used classroom teachers to perform the interventions that naturally were related to educational interventions. About 30% of the studies used parents or “families” for interventions at some level, beyond simply an explanation of the project and getting consent for their children. Nutritionists were involved at some level of direct intervention in 16% of the studies and were used in a few studies to train classroom teachers to deliver health‐enhancing messages. Three studies involved the family pediatrician. Researchers were involved in two studies. About four studies involved several groups, giving a community awareness education focus. A few projects used unique interventionists, such as Rosi et al. (2016b) study that used a humanoid robot for dietary education; Sotgiu et al. (2009) using Cub Scout leaders; and Centis et al. (2012) using university faculty for health‐enhancing messaging.

### 3.8 Research Designs Given Purpose, Theoretical Model, Interventions, and Assessment

Naturally, the research design is foremost driven by the purpose of the project. As noted in the early Findings section for Table 2, the major purpose of the studies found for this scoping review of school‐based nutrition education programs in Italy during this half‐century timeline was to engender a “healthy lifestyle” early on, which included healthy food choices and ample physical activity. A secondary purpose was to learn about how to reduce overweight or obesity in children with educational and physical activity programs in schools. The interventions aimed for outcomes that improved either the knowledge and/or behavior of eating habits of youth in the studies and at a lesser level for youth to develop a positive attitude toward healthy food consumption and/or helpful physical activity. There were a few projects that could qualify as skill building, such as food preparation for parents (Centis et al. 2012; Franceschi et al. 2021; Grassi et al. 2016) and students exposed to social marketing campaigns to be alert for positive or negative messages on eating habits (Nosi et al. 2021).

Furthermore, a purpose of 10 of these studies, of the 42 reviewed, continued evaluating recognized “brand name” nutrition education programs or campaigns, and some combined this with physical activity programs for youth. These studies aimed to continue validating their use with youth, increase the number of subjects participating, or expand the use of the program to other age groups. The following branded programs used in the projects found in this scoping review are as follows:

*School Fruit* (Roccaldo et al. 2017; Scuderi et al. 2016): The EU “School Fruit” scheme, later combined with the vegetables and milk scheme, aims to promote awareness of a healthy diet among children and adolescents in schools or other formal educational organizations. The programs, conducted by a range of educators, aim to develop healthy food habits early on if possible and enculture a sense of taste for healthy foods, and have students gain an understanding of how diet, growing and production of food, agriculture, and the environment are interconnected.

*Italian “Eat Project”* (Ermetici et al. 2016) is a school‐based multicomponent dietary program that combines environmental changes (such as alternative healthy food choices in vending machines and healthy use of water bottles) and educational interventions (such as classroom lessons, educational posters, focused textbooks, and individual reinforcement tactics), for students to maintain a healthy body weight.

*MaestraNatura* (Scazzocchio et al. 2021; Varì et al. 2022) is an innovative nutritional education program aimed at promoting a healthy lifestyle in first‐level secondary school students during a multiyear intervention. Topics covered include learning about food items, nutrition, healthy eating, and a sustainable diet, integrating into the school curricula in order to facilitate teachers in their work.

*Maìna Fairy Project* (Trento et al. 2012) aims for “education for healthy growth” of preschoolers who learn about correct eating. It uses games to promote correct food choices and balances meal and physical development in children 3–5 years old.

*Kaledo* (Amaro et al. 2006; Viggiano et al. 2015; Viggiano et al. 2018) is a board game for nutrition education of children and adolescents. Using a mix of food and activity cards and moving around the board, players, after learning their height, body structure, and current weight, try to balance their total energy supply and expenditure to reach their ideal weight, given their height and body structure.

*GiochiAMO* (D'Egidio et al. 2021) is a health promotion program that uses knowledge‐based education and games to teach healthy nutrition and physical activity among children (6–8 years old).

*Shake Your Health* project (Franceschi et al. 2021) is an integrated and comprehensive model to prevent and treat overweight and obesity in low socioeconomic status and minority groups living in three different districts in the north of Italy. An app and a cookbook promoting transcultural nutrition and a healthy lifestyle were developed, and no‐cost physical activities were organized.

*IDEFICS* (Haerens et al. 2009), which is “identification and prevention of dietary‐ and lifestyle‐induced health effects in children and infants,” implements a standardized community‐based multicomponent healthy eating intervention for younger children in eight different countries.

*Giocampus* (Rosi et al. 2016a; Vanelli et al. 2014) is an educational project that aims to promote the well‐being of youth through a program of physical activity and healthy eating education. The school lessons use a “learning through playing” approach to teach children about nutrition. There is an annual summer sports school at the university. It also teaches environmental sustainability, and students engage in a range of creative activities to promote healthy lifestyles.

*Food Dudes Health Eating* program (Presti et al. 2015) is a behavior change program based on three core principles: (1) role modeling, (2) rewards, and (3) repeated tasting of healthy foods, most notably fruits and vegetables.

Thus, and as noted in the prior results section, most of the research undertaken in this area was not theory driven, but used a health promotion model (62%), therefore research designs were mostly experimental focusing on what students, for the most part, “said about knowledge and behavior change” via self‐administered questionnaires (what they now know and what they are doing). Some studies involved direct observation and anthropometric measures by researchers.

Data techniques used for knowledge acquisition of healthy food “that are good for you” or behavior changes that improved healthy food choices were 15 studies that used questionnaires for behavior change in food consumption, 13 studies used questionnaires to show increased in healthy food knowledge, and 10 studies used food consumption diaries. Most of these three data techniques were self‐administered by youth subjects, except for the very young subjects wherein the parents or teachers assisted in answering the survey or writing in a diary. A few studies involved changes in parent and overall family behavior.

As noted earlier in an earlier results section above, about 14% of the studies combined a significant focus on physical activity with a dietary message. Six of the studies used self‐administered questionnaires or diaries to measure changes in physical activity. Nineteen studies had an anthropometric measure. These included 11 studies that measured body mass index, four that measured the height and weight of students, two that measured just the weight of students for changes, one study that took a urine analysis of subjects to measure iodized salt intake, and one study that measured students blood pressure and cholesterol. One study conducted standardized motor evaluation tests of students.

### 3.9 Anthropometric Outcome Changes

Nineteen of the studies in this scoping review took an anthropometric measure of their subjects. Of these, six studies did not look for outcomes of the anthropometric measure taken but used these for another purpose, such as markers for comparisons in a nutrition education program, to randomize subjects into groups, or serve as a rationale for undertaking the educational program presented, that is, motivate buy‐in to get school administrators or parents to have students participate in a proposed intervention.

The “N” for outcomes is larger than the number of studies, given some studies had more than one anthropometric measure. Stakeholders with an interest in behavioral and physical changes for school students should be optimistic about the state of programs available for students to “get on the right track” in maintaining ideal body weight or participating in physical activity, given that 15 studies showed positive changes in anthropometric outcomes, compared with three (negative) outcomes and two outcomes with no change.

Studies undertaken by Angelico et al. (1991), Franceschi et al. (2021), and Gallotta et al. (2016) had negative outcomes. The former, one of the earliest studies, learned that “school‐delivered programs of general nutrition education for the control of risk factors does not appear to be able to control child obesity.” Franceschi et al. (2021) study was not able to significantly decrease the BMI *z*‐score at an obesity clinic. Gallotta et al. (2016) found that body fat mass % increased after intervention in all groups: experimental Group 1 versus experimental Group 2 versus control group after three 5‐month combined PE and nutrition education programming in school.

Reviewing the “Conclusions” of 13 of the studies having anthropometric outcomes, it is not surprising to learn that in order to reach a significant and long‐standing outcome in battling the overweight and obese problem in Italy, programs should involve a large number of youths, for a “the longer the better” approach, and apply more than a single standard intervention, that is, use educational programs with environmental controls—and even better use social marketing strategies at a broad community level. It is not clear if giving information to students and families about the weight status of students changes the trajectory of students' weight gain. Little had been done to provide incentives to families and subjects in these programs. There is much enthusiasm from one lead author of two studies (Viggiano et al. 2015; Viggiano et al. 2018) that the board game Kaledo, which provides nutrition education for children and adolescents at school, can have positive results in mitigating or keeping students from becoming obese at any grade level.

### 3.10 Dietary Knowledge and/or Behavior Outcome Changes

Stakeholders with an interest in dietary behavioral changes for school students should be optimistic about the state of programs available for students to “get on the right track” in beginning and maintaining a healthy diet, given that there were 33 positive changes in the 35 studies that measured dietary behavior change. Only four studies showed no change in a dietary outcome measure for students' behavior. Two projects had a mix of positive and no change outcomes. All eight of the recognized “brand name” nutrition education programs as mentioned above in the opening design section of the “Findings” had positive behavior changes among subjects. Two of the 10 projects did not have individual outcomes measures, as the researchers looked at organization *Fata Maìna* project (Trento et al. 2012) and group preferences [identification and prevention of dietary‐ and lifestyle‐induced health effects in children and infants (IDEFICS)] of the programs' implementation (Haerens et al. 2009).

As noted in the early findings section, few studies of the 42 reviewed used health education theory‐driven research. By far the most common approach was a health promotion model. It appears that the investigators were confident that they had enough information about “what works” in program objectives, design, and implementation in promoting healthy eating habits in youth. In this regard, one overall observation for approaches to dietary and nutritional programs evaluated in this review is the research teams using a three‐step approach to *educate, demonstrate*, and *practice* healthy eating. Many of the studies had all three components to first provide information in some type of knowledge exchange or discovery, then demonstrate healthy food choices and consumption, followed by practice at home (breakfast, dinner, and snacks) or during lunchtime or snack time in school.

In short, the research shows that while short‐term and didactic approaches to teaching a healthy lifestyle will produce some behavior change, these changes are unlikely to last. Programs that involve multicomponent approach of educating, demonstrating, and practicing, and involve more influencers (i.e., teachers and parents) will have more positive and longer‐term behavior changes.

Not surprisingly, there is enthusiasm for community‐based (Tripodi et al. 2011) nutrition programs and social marketing (Nosi et al. 2021) approaches. However, these take more resources and pose challenges to evaluate. Few studies of the 42 used this approach to provide enough formative and summative evaluations to be meaningful.

Few studies used environmental changes in the interventions. Direct and close changes to food consumption behavior would naturally take place with any school‐provided food, such as breakfast, snack, or lunch programs. Lazzeri et al. (2013) and Ermetici et al. (2016) showed positive behavior with students selecting food from vending machines in school. Changes to the home environment are driven by parents, especially for the younger students. There are a healthy number of studies among the 42 in this review that used parental participation at some level, and some with changes to food consumption at home to inform further students. Research teams did not venture much into studying other places outside of school and home, aside from one study that worked with Cub Scouts (Sotgiu et al. 2009) and (Tripodi et al. 2011) involving sporting clubs and Vanelli et al. (2014) working with a university sports school to promote physical activity and a healthy lifestyle.

### 3.11 Study Effect on Other Assessed Health Attitudes and Behaviors Outcomes

Ten of the 42 published studies in this review can be described better as “other” knowledge, attitudes, or behavior changes aside from the main focus of nutrition and physical changes from dietary or physical activity interventions.

Trento et al. (2012) used various games to promote the psychophysical development of the child, which in turn promoted healthy eating. Latino et al. (2023) measured changes in building the self‐efficacy of subjects. A couple of studies did not look at physical activity per se but measured sedentary time of subjects. The remaining projects involved parents, public officials, or groups in parts of the project to increase some type of collaboration on the development of the students toward a healthy lifestyle.

## 4 Discussion

This scoping review involved 35 years of research published about school‐based nutrition education interventions (1990 to April 2024), thus providing a robust look at the nature and extent of this important area of child and adolescent health promotion undertaken in Italy. The major purpose of the studies found for this scoping review was to engender a “healthy lifestyle” early on that included healthy food choices and ample physical activity, which in turn would avoid or mitigate the rise in the overweight and obesity problem among youth in this country.

Stakeholders with an interest in dietary/nutrition behavioral and physical activity of children and adolescents in Italy should be optimistic for the state of programs available for students to “get on the right track” in practicing good nutrition and participating at some level of physical activity given we found that: (1) a robust number of studies (*N* = 42) have been conducted to test the effectiveness of dietary/nutrition programs in schools during this almost half‐century review; (2) projects have been undertaken by relatively large number of investigators, demonstrating a high level of collaboration, as the average number of investigators on a publication is 8.6; (3) the lead authorship is spread among the 42 studies with only a few repeat lead authors, which shows a deep level of expertise in this area of research; (4) projects involved all three sectors with the nonprofit and government sector the most evident; (5) the outcomes for both dietary behavior change and anthropomorphic changes after an intervention have been by far positive after interventions; (6) the interest in this research area has accelerated after the 1990s. This longitudinal review coupled with the six characteristics above likely demonstrates a high level of reliability of interventions in this area of study.

In looking at an important outcome when reviewing work in this area, as mentioned in the Methods section: “Do children, adolescents, parents, or school staff members in Italy (P) that are offered school‐based nutrition interventions (I) have improved health and wellness parameters (O) compared with those that do not participate in school‐based nutrition interventions(C),” we look at the controlled trials. Ten studies used randomized controlled trials, and seven studies used controls but did not randomize the subject groups. Impressively, all these studies showed positive results in behavior change by subjects after interventions.

Of the 35 studies that measured dietary behavior change, 33 reported a positive change. Two projects had a mix of positive and no change outcomes. Similarly impressive outcomes are the studies that made anthropometric measures of students. Fourteen studies showed positive changes in anthropometric outcomes, compared with three (negative) outcomes and two outcomes with no change.

The purpose of 10 of the studies undertaken involved evaluating recognized “brand name” nutrition education programs or campaigns, and some combined this with physical activity programs for youth, which shows schools at all levels in the country have a healthy number of “off the shelf” education programs to use to meet their dietary/nutrition objectives for students. Furthermore, all 8 of the 10 that had outcome measures showed positive behavior change among students.

About two‐third of the projects used manageable research designs, such as single‐group pretest and posttest, and many used survey instruments to learn about knowledge and behavior changes among subjects after interventions. However, when working with youth, even simple designs create challenges given the need for school administrator and teacher approval and cooperation, informed consent from parents, and cooperation from parents.

Investigators in many of the studies use the successful steps to teaching behavior change as *educate, demonstrate, and practice*. Using all three steps does bring challenges to designing longer interventions and follow‐ups. At the end of the articles and “in conclusion” it may be interesting to note that few used what is often an “obligatory” comment that “further research is needed” in the phenomenon under study and with select subjects. This may signal confidence in findings thus far in this area of research in the country.

### 4.1 Research Gaps for Improving Dietary Knowledge and Anthropometric Changes

There are gaps in types of interventions that should stimulate more research in this area. Few studies are using social marketing techniques that on the one hand expand those and what is involved in an intervention but relieve the intensity needed for narrow interventions such as didactic approaches by teachers. Related to this, but narrower, is the opportunity to use more environmental controls where students live, learn, play, socialize, and pray. For both approaches mentioned, there is evidence that treating this as a “public health problem” should provide more traction for those concerned. In fact, the social marketing approach could be more beneficial to the adolescent population who are not well studied during this half‐century time.

Children only were the most studied, involved in 31 of the 42 studies, while six studies involved adolescents alone and five studies involved both youth groups. This review shows that opportunities for more insight into working with adolescents are evident, especially knowing the success environmentally focused interventions can have on youth behaviors in other areas of unhealthy consumption such as tobacco and alcohol. Investigators gave little information about the characteristics of the students, aside from grade level, which could be significant information to know about the many subjects used in these studies.

Investigators seem to have good cooperation from schools, teachers, and parents; however, the articles provide little formative evaluation, which could be helpful in designing further studies and recruiting schools and parents to allow interventions for students.

Most studies involved a Health Promotion Model. More can be done in working with theory‐driven projects. Given one goal in working with students is to instill healthy lifestyle habits at a young age to avoid being overweight, more can be done to provide interventions that have multicomponents, not simply didactic interventions.

What could be the biggest opportunity to “fill a gap” for those interested in making change in the youth overweight and obesity problem is to heed the advice of some public health professionals and groups to “work upstream” on our healthcare and public health problems. Much is done in the downstream, and consequent mitigating needs, and such downstream efforts are popular to public policymakers as they make for “few waves” in the status quo. Few public policymakers will expend political capital to attack a problem by going upstream to see “who or what is pushing people into the unhealthy river,” in which they suffer (Gambescia 2024, p. 49).

### 4.2 Limitations

One often limitation reported when working with human subjects is caution for the readers to generalize findings, given the low number of subjects studied. It is fair to say that much of the research in this area during this almost quarter century has involved a useful number of subjects to make recommendations. Some of the studies have reached into the thousands. While we noted challenges to working with youth in schools, once approved for study, school‐delivered programs are quite efficient in getting a large number of subjects, as the students are assembled in relatively large groups. Studies with a small number of subjects noted the limitation of generalizability.

Using survey questionnaires and diaries to measure student eating and physical activity behavior has historically been a limitation for accuracy, given that most people, including youth, will underreport unhealthy behavior and overreport healthy behavior (Connor 2020). Added to this limitation is a sense that the status of youth reporting to authority, so to speak, such as a parent or teacher, leads students to report favorable responses to questions. Some studies had a third party assisting in student responses to questions that could cause bias in the responses. Many investigators seemed confident in the number of subjects recruited; however, a limitation in many studies is not having a long enough follow‐up period, as the investigators had to work within the time parameters of the school calendar.

Studies using a scaffolded training program for those who deliver a health‐enhancing message to subjects raise questions of variability in the delivery of the message. Studies needed to work closely with other groups rather than directly with the subjects (administrators, teachers, parents) which can challenge data collection integrity. Little information was given on summative measures to measure the integrity of delivering a protocol to students under study. Not all articles gave a substantive report on the studies' limitations. Finally, there were limitations unique to a study, such as instruments used for anthropometrics, homogeneity of subjects, and providing a lot of information in a short amount of time.

## 5 Conclusion

This scoping review highlights the effectiveness of school‐based nutrition interventions in Italy in fostering healthy dietary behaviors and mitigating overweight and obesity among children and adolescents. The findings indicate that programs employing a multicomponent approach encompassing education, demonstration, and practice are most effective in producing positive and sustained behavior changes. In other words, studies that had this three‐step process of (1) providing knowledge of “good nutrition” and the importance of healthy eating, (2) followed by some type of demonstration by the instructors, such as taste testing new foods, (3) followed by short to midterm practice of what is eaten at school and home had the best outcomes. These programs often leverage Italy's rich culinary traditions, particularly the Mediterranean diet, as a foundation for promoting healthy eating habits.

While the reviewed studies demonstrate a robust body of evidence supporting the benefits of school‐based interventions, significant gaps remain. Notably, there is limited focus on adolescent populations, insufficient use of social marketing techniques, and underutilization of environmental approaches. While adolescence is a turbulent time and teens gravitate toward risky behaviors, their behaviors are malleable. This changeability can be seen as a positive as teens do not have ingrained habits and may be open to “trying new things.” To engage in conversations about health nutrition and to instill healthy behavior in teens, today's teachers, health promotion specialists, and parents need to “get in the way” of how teens are “curating a steading stream of information,” especially competing against digital media (Robertson 2022). These gaps present opportunities for further research and program development.

To enhance the impact of school‐based nutrition programs, there is a need for stronger collaboration between schools, families, communities, and policymakers. While considering the range of stakeholders can be seen as untenable, there is much literature demonstrating the success and “turn‐key” resources in building coalitions to achieve health enhancement goals in a community. Certainly, there is a host of challenges in coalition building and its implementation of programs that provide tangible outcomes, yet this can be balanced against the high salience level improving the health of the youth can enjoy, especially among policymakers (Kingdon 2014; Nagorcka‐Smith et al. 2022). A more comprehensive application of theoretical models, such as the Health Promotion Model, alongside regionally tailored and culturally sensitive strategies, will help to address the diverse sociocultural influences across Italy. Coordinated efforts at the national, regional, and local levels are essential to overcoming barriers, such as limited resources, variability in implementation, and external challenges. By addressing these gaps and challenges, school nutrition interventions in Italy can further contribute to promoting healthy lifestyles, improving health outcomes, and supporting the development of lifelong healthy eating habits among children and adolescents. These findings provide valuable insights for stakeholders aiming to strengthen and expand the reach of these critical health promotion efforts.

## Author Contributions

**Stephen Gambescia:** formal analysis (lead), investigation (equal), methodology (equal), project administration (equal), resources (equal), software (equal), supervision (equal), validation (equal), visualization (equal), writing – original draft (equal), writing – review and editing (equal). **Basil H. Aboul‐Enein:** conceptualization (lead), data curation (lead), investigation (lead), methodology (lead), project administration (lead), resources (lead), software (lead), supervision (lead), validation (equal), visualization (equal), writing – original draft (equal), writing – review and editing (equal). **Teresa Keller:** writing – original draft (equal), writing – review and editing (equal). **Fatmah Almoayad:** writing – original draft (equal), writing – review and editing (equal). **Nada Benajiba:** resources (equal), validation (equal), writing – original draft (equal), writing – review and editing (equal).

## Ethics Statement

The authors have nothing to report.

## Conflicts of Interest

The authors declare no conflicts of interest.

## Supporting information


Data S1.

## Data Availability

No data were used for the research described in the article.
