# Supplementary material for: Effectiveness of School‐Based Nutrition Interventions in Italy: A Scoping Review
Source: Food Sci Nutr. 2025 Jul 15;13(7):e70266. doi: 10.1002/fsn3.70266 (PMC12264323; doi:10.1002/fsn3.70266)
Supplement: Supplementary file 1 — Data S1. [file FSN3-13-e70266-s001.docx]

**Supplementary material**

“school” [All Fields]; “school-based” [All Fields]; “elementary” [All Fields]; Primary” [All Fields]; “secondary” [All Fields]; “nutrition” [All Fields]

AND

“Intervention” [All Fields]; “Program” [All Fields]; “Education” [All Fields]

AND

“Italy” [All Fields]

***Italian Translation***

*Scuola; basato sulla scuola; elementare; Primario; Secondario; nutrizione* [All Fields]

*AND*

*Intervento; Programma; Educazione* [All Fields]

*AND*

*Italia* [All Fields]
